# High Detectivity of PbS Films Deposited on Quartz Substrates: The Role of Enhanced Photogenerated Carrier Separation

**DOI:** 10.3390/s23208413

**Published:** 2023-10-12

**Authors:** Quanjiang Lv, Rongfan Li, Liangchao Fan, Zhi Huang, Zhenyu Huan, Mingyang Yu, Haohua Li, Guiwu Liu, Guanjun Qiao, Junlin Liu

**Affiliations:** 1 School of Materials Science and Engineering, Jiangsu University, Zhenjiang 212013, China; lvquanjiang@ujs.edu.cn (Q.L.);; 2 Key Laboratory for Theory and Technology of Intelligent Agricultural Machinery and Equipment, Jiangsu University, Zhenjiang 212013, China

**Keywords:** lead sulfide films, infrared, photodetectors, sensitization mechanisms

## Abstract

PbS films grown on quartz substrates by the chemical bath deposition method were annealed in an O_2_ atmosphere to investigate the role of oxygen in the sensitization process at different annealing temperatures. The average grain size of the PbS films gradually increased as the annealing temperature increased from 400 °C to 700 °C. At an annealing temperature of 650 °C, the photoresponsivity and detectivity reached 1.67 A W^−1^ and 1.22 × 10^10^ cm Hz^1/2^ W^−1^, respectively. The role of oxides in the sensitization process was analyzed in combination with X-ray diffraction and scanning electron microscopy results, and a three-dimensional network model of the sensitization mechanism of PbS films was proposed. During the annealing process, O functioned as a p-type impurity, forming p^+^-type PbS layers with high hole concentrations on the surface and between the PbS grains. As annealing proceeds, the p^+^-type PbS layers at the grain boundaries interconnect to form a three-dimensional network structure of hole transport channels, while the unoxidized p-type PbS layers act as electron transport channels. Under bias, photogenerated electron–hole pairs were efficiently separated by the formed p^+^-p charge separation junction, thereby reducing electron–hole recombination and facilitating a higher infrared response.

## 1. Introduction

Lead sulfide (PbS), with a rock salt structure, plays an important role in group IV–VI compound semiconductors. PbS is considered to be a characteristic semiconducting material with a narrow bandgap (0.41 eV at 300 K) and a large exciton Bohr radius (18 nm) [1]. These features make PbS widely used in infrared photodetectors [2,3], solar cells [3], gas sensors [4,5], and other fields. In particular, PbS polycrystalline photoconductor detectors for near- and mid-infrared applications are sought after due to their numerous advantages, encompassing excellent photoresponse, high detectivity, swift response time, and cost-effectiveness. Various deposition techniques have been employed for PbS-based infrared (IR) detectors, including chemical bath deposition (CBD), vacuum thermal evaporation, chemical vapor deposition (CVD), molecular beam epitaxy (MBE), magnetron sputtering, etc. [6,7,8,9]. Among these methods, CBD stands out as the most widely used method for the fabrication of PbS films.

The as-grown PbS films are insensitive to IR response; thermal annealing (also known as sensitization) at different temperatures and atmospheres is required to improve their IR photoelectric response [10,11,12,13,14]. Despite the fact that the mechanism of sensitization of PbS films is still highly controversial in the academic community, the effect of oxygen on the performance enhancement of thin PbS films has been unanimously recognized [11]. Oxygen has long been considered to be the key sensitizing element, acting as an effective sensitization promoter through defect passivation and the formation of oxide layers at the grain boundaries during the sensitization process at higher temperatures in an oxygen environment [15,16,17]. In addition, oxygen serves as a p-type trap, effectively reducing carrier recombination and, thus, prolonging the carrier lifetime and enhancing the photoelectric detection capabilities of PbS [18]. Several models have been proposed to elucidate the optoelectronic sensitization of PbS thin films, including the barrier model [15], the generalized model [16], the minority carrier trap model [19], and the charge separation model [10]. Amongst these, the charge separation model as proposed by Qiu et al. posits that after sensitization, PbS grains randomly interconnected to form p-type conducting channels, while oxide layers between the grain boundaries are interconnected to form n-type conducting channels. Under bias, photogenerated electron–hole pairs are effectively transported by the formed conductive channels, reducing electron–hole recombination and consequently enhancing the photoelectric response [10]. Up to now, although numerous studies have been conducted on annealing in an oxygen-containing atmosphere, such research seems to have reached a bottleneck. Therefore, it becomes imperative to find a suitable sensitization mechanism to guide the improvement of the response of PbS detectors to IR radiation.

In this work, the as-grown PbS films prepared by the CBD method were sensitized at different temperatures in an oxygen atmosphere to gain deeper insights into the sensitization mechanisms responsible for inducing the IR response. The X-ray diffraction (XRD) results showed that as the annealing temperature gradually increased from 400 °C to 700 °C, there was a gradual intensification in the peaks associated with oxide phases. Additionally, the field-emission scanning electron microscopy (FESEM) results revealed an observable increase in the surface grain size. This phenomenon can be attributed to the heightened oxygen penetration capacity at higher temperatures, resulting in the oxidation of PbS. To further determine the role of the number and morphological distribution of oxides in the PbS sensitization process, it was observed that the oxides were connect to one another after PbS etching, forming a 3D interweaving network structure. Based on the microstructural evolution, composite phase, and photoelectric changes under various sensitization processes, a p^+^-p 3D network transport model was proposed to elucidate the charge separation mechanism of PbS IR detection.

## 2. Experimental Section

PbS films were grown on quartz substrates by the CBD method. The reaction solution was composed of lead acetate [Pb(CH_3_COO)_2_·3H_2_O], trisodium citrate (C_6_H_5_Na_3_O_7_), potassium hydroxide (KOH), and thiourea (CH_4_N_2_S) at a concentration ratio of 1:2:5.7:2. The cleaned quartz substrates were immersed in the deposition bath at 80 °C for 4 h. After deposition, the samples were rinsed with deionized water and dried. The as-grown PbS films were uniform, mirror-reflective films (named S0), which were subsequently annealed in pure oxygen at 400, 450, 500, 550, 600, 650, and 700 °C for 1 h (named S400, S450, S500, S550, S600, S650, and S700, respectively). The Cr/Au (50 nm/200 nm) electrodes were deposited by magnetron sputtering. The distance between the two electrodes was 1 mm, and the width of the electrodes was 200 µm (active area = 1 × 1 mm^2^).

The phase and crystal structures of these samples were characterized by XRD using Cu Kα radiation (Rigaku Ultima IV, *λ* = 1.5406 Å) in the 2*θ* scan range with a step size of 0.02° in the range of 10° to 80°. The surface morphology and grain size of the samples were observed by FESEM (Nova Nano450, FEI, Hillsboro, OR, USA). The film thickness was measured using a step profiler (Dektak 150, Veeco, New York, NY, USA). Photoelectric properties were evaluated using a photoelectric test system with a source meter (Keithley 2450, Keithley Instruments, Solon, OH, USA) connected to a probe station at room temperature, and the incident laser (*λ* = 1550 nm) was used as an excitation source with a waveform generator (Rigol DG821, Rigol Technologies, Suzhou, China) to control the switching of the laser.

## 3. Results and Discussion

**Figure 1:**
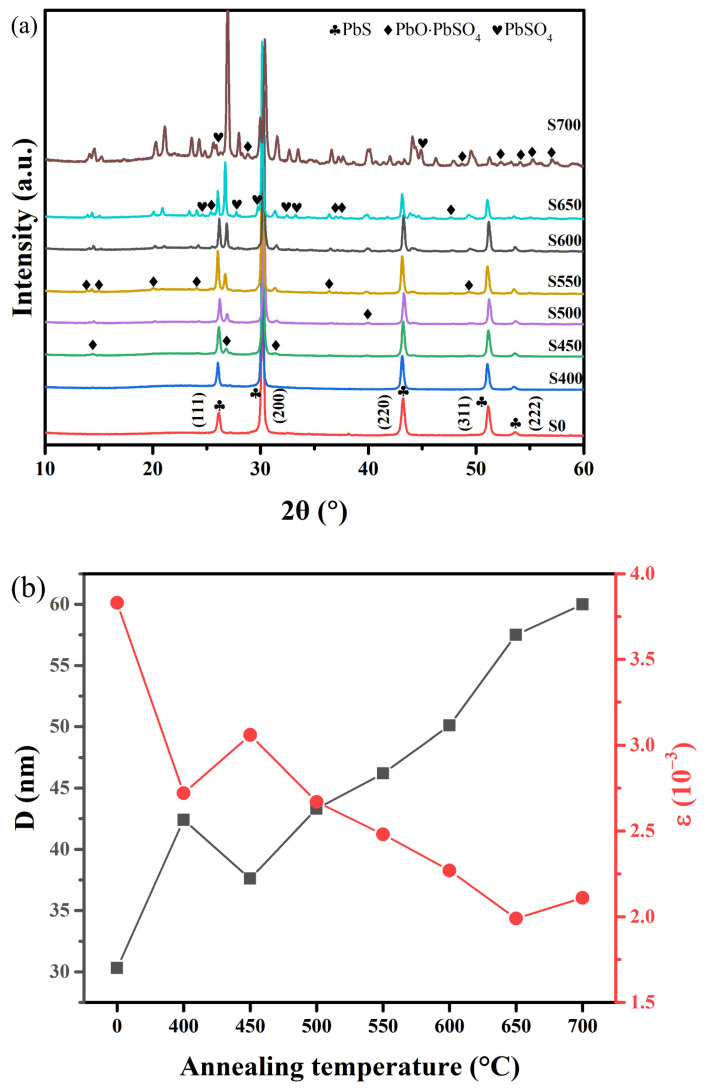
(**a**) XRD patterns of samples S0 to S700. (**b**) Average grain size and average microstrain with annealing temperature for samples S0 to S700.

XRD tests were used to reveal the crystal-phase evolution of PbS films deposited on quartz substrates and annealed at different temperatures. The XRD patterns are shown in Figure 1a. The diffraction peaks of the as-grown film of sample S0 were calibrated by the phase-pure cubic structure of the space group *Fm3m* and consistent with the standard JCPDS data (PDF#05-0592), indicating the high purity of CBD-PbS films. Intriguingly, for sample S400, it can be seen from the diffraction patterns that after undergoing O_2_ sensitization at 400 °C for 1 h, no diffraction peaks were observed for any oxide phases or other new phases apart from the PbS phase. The absence of the oxide phases was probably due to the relatively lower sensitization temperature, resulting in limited oxide production, falling below the XRD detection limit [20]. As the annealing temperature increased to 450 °C, three small peaks at 2*θ* = 14.3°, 26.7°, and 31.4° could be identified for sample S450, corresponding to the (200), (310), and (020) planes of the PbO·PbSO_4_ phase, respectively. As the annealing temperature continued to increase to 500~600 °C, other diffraction peaks of the PbO·PbSO_4_ phase were gradually observed, and the intensity of the diffraction peaks of the PbO·PbSO_4_ (310) plane gradually increased with the increase in the annealing temperature, indicating that the crystallinity of the PbO·PbSO_4_ phase improved. As the temperature increased to 650 °C, the diffraction peaks corresponding to the PbSO_4_ phase began to appear, and the intensity of the diffraction peaks of the PbO·PbSO_4_ (310) plane increased further. When the temperature was increased to 700 °C, a significant intensification in the diffraction peaks associated with oxide phases was noted, suggesting the potential generation of a substantial quantity of oxide phases in sample S700. The average crystal size (*D*) and average microstrain (*ε*) were calculated using the following equations [21]:

(1) $$D=\frac{0.9\lambda }{\beta \cos\;\theta }$$

(2) $$\;\varepsilon =\frac{\beta }{4\tan\;\theta }$$

where λ is the incident X-ray wavelength (λ = 1.5406 Å), while *β* and *θ* represent the full width at half-maximum and the Bragg diffraction angles of the peaks, respectively. As shown in Figure 1b, the average grain size of S400 was significantly larger compared to S0. This enlargement resulted from the recrystallization of small grains into larger ones under the thermodynamic conditions created by high temperature [22]. Continuing to increase the annealing temperature to 450 °C, the grain size of the PbS films began to decrease. This reduction can be attributed to the oxidation of the PbS grains, leading to the formation of the new PbO·PbSO_4_ phase, as depicted in Figure 1a; this, in turn, reduced the grain size. With continued temperature increase, the PbO·PbSO_4_ phase that formed on the surface of the PbS films inhibited further oxidation of the internal PbS [23]. Simultaneously, the internal PbS grains that did not react with O_2_ also recrystallized at high temperatures, resulting in an overall increase in the average size of the PbS grains. The results of the average microstrain with annealing temperature for eight samples indicated that annealing can significantly reduce the average microstrain of PbS films grown by the CBD method, which is consistent with the results reported by Motlagh et al. [24]. Notably, the average microstrain of the PbS film increased abruptly when the annealing temperature increased from 400 °C to 450 °C, probably due to the generation of the oxide phases, which is consistent with the results of the XRD test shown in Figure 1a. Surprisingly, as the annealing temperature increased from 650 °C to 700 °C, the average microstrain of the PbS films increased rather than decreasing, possibly owing to excessive oxide generation in sample S700 at this temperature.

**Figure 2:**
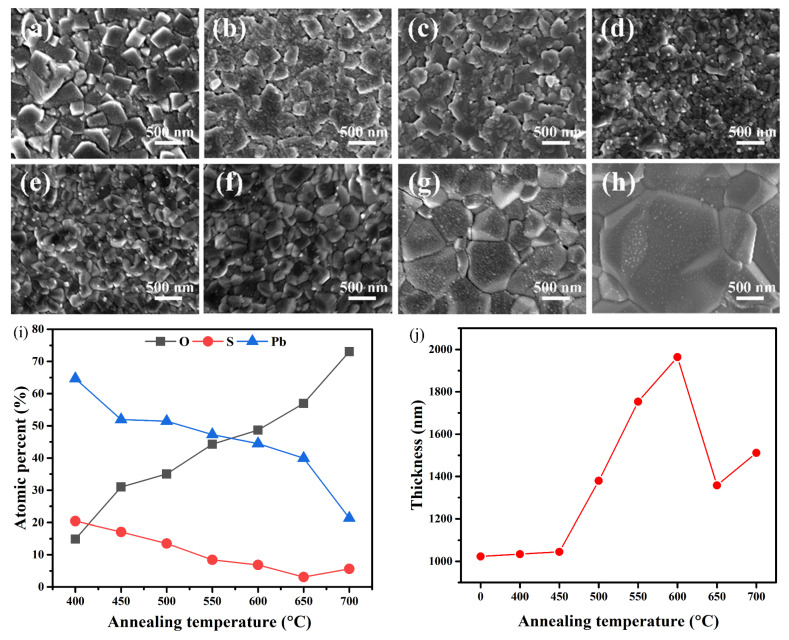
FESEM images of the as-grown and annealed PbS films: (**a**) As-grown, and annealed at (**b**) 400 °C, (**c**) 450 °C, (**d**) 500 °C, (**e**) 550 °C, (**f**) 600 °C, (**g**) 650 °C, and (**h**) 700 °C. (**i**) Atomic ratio of O, S, and Pb for samples S400 to S700. (**j**) Thickness variation of samples S0 to S700.

Figure 2a displays the surface morphology of the as-grown sample, which consists of congregating cubic PbS microcrystals of different sizes with smooth surfaces. The microstructural evolution of the films annealed at temperatures ranging from 400 °C to 750 °C for 1 h is depicted in Figure 2b–h. As shown in Figure 2b, after annealing at 400 °C, the grains still appear as compact cubic microcrystals, but the surface has become less smooth and covered by some smaller grains, indicating that the PbS film has been oxidized under this temperature. Nevertheless, the XRD pattern of sample S400 in Figure 1a does not exhibit any diffraction peaks corresponding to the oxide phases, possibly due to its low content and poor crystallinity. As the annealing temperature was further increased (Figure 2c–h), numerous pebble-like grains emerged on the surface, and their size gradually increased with the increase in the annealing temperature. The largest grain size, exceeding 2 μm in diameter, was observed at 700 °C, as shown in Figure 2h.

To further analyze the composition of the irregular structure on the surface of the samples, X-ray energy spectroscopy (EDS) analysis was performed on these samples, and the results are shown in Figure 2i. As depicted in Figure 2i, the atomic ratio of O in the PbS films grown on quartz substrates gradually increased with the increase in the annealing temperature, while the atomic ratios of Pb and S decreased. The increase in the atomic ratio of O at high temperatures indicates that although the newly formed oxide phase can inhibit further oxidation of the PbS films, higher temperatures can enhance the oxygen diffusion depth, leading to further oxidation of the PbS films. Figure 2j shows the results of the thickness variation of the as-grown film and samples at different annealing temperatures. When the annealing temperature was below 450 °C, the film thickness remained almost constant. When the annealing temperature was between 450 °C and 600 °C, the film thicknesses gradually increased with the increase in the annealing temperature, probably due to the formation of the PbO·PbSO_4_ phase. The film thickness significantly decreased when the annealing temperature surpassed 600 °C, possibly due to extensive film sublimation in this temperature range. Kamchatka et al. [25] also reported that annealing temperatures above 577 °C resulted in the sublimation of PbS. While the newly generated oxide phase contributes to increased thickness, it fails to compensate for the thickness reduction caused by film sublimation at these temperatures.

**Figure 3:**
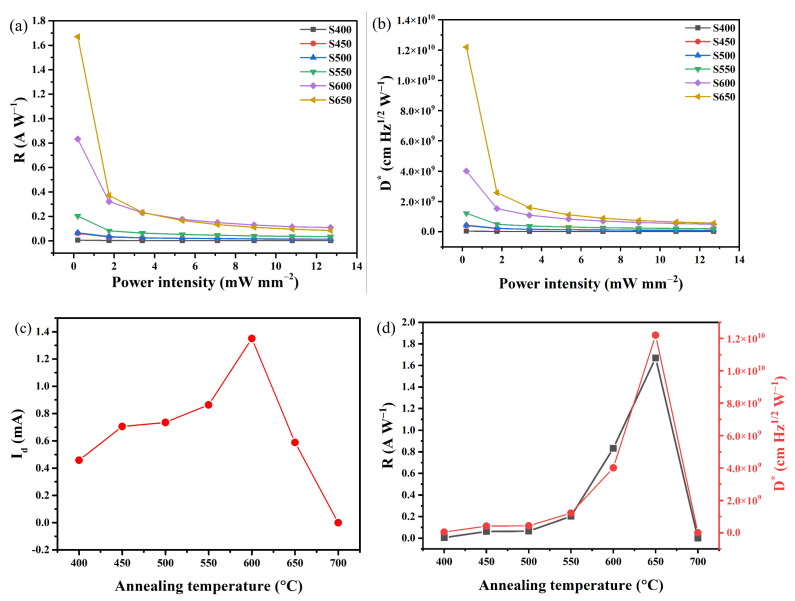
Plots of (**a**) *R* and (**b**) D* curves for the PbS detectors as a function of incident optical power. (**c**) *I_dark_* curves of PbS thin films at different annealing temperatures. (**d**) Plots of *R* and D* curves for the PbS detectors at different annealing temperatures.

The photoelectric detection performance of sensitized PbS infrared photoconductive detectors was determined by measuring the device’s photocurrent, and the results are shown in Figure 3. To evaluate the performance of these sensitized PbS detectors, we measured their responsiveness and detectability at various incident laser powers (λ = 1550 nm, modulation frequency: 1 Hz) and bias voltages (50 V). The photoresponsivity (*R*) is defined as $R=I_{ph}/P$, where *Iph* is the photocurrent and *P* is the is the incident laser power. The detectivity (D*) is defined as $D^{*}=R\times \sqrt{A}/\sqrt{2qI_{dark}}$, where *A* is the sensitive area of the photodiode, *q* is the absolute value of the electron charge (1.6 × 10^19^ C), *I_dark_* is the dark current, and *R* is the responsivity [26]. As shown in Figure 3a,b, it is evident that both the *R* and D* values for sensitized PbS detectors decrease with increasing *P* values. This decrease may be attributed to the elevated scattering and recombination rates of hot carriers at higher incident powers [27], thereby reducing the conversion efficiency of photogenerated carriers to photocurrent. This trend is consistent with findings observed in prior experiments conducted by Liu and Hou et al. [28,29].

To further investigate the effect of annealing temperature on the photoelectric properties of PbS thin films grown on quartz substrates, the *I_dark_*, *R*, and D* values of PbS films annealed at different temperatures at a low incident power density (*P* = 0.2 mW mm^−2^) were characterized according to the results shown in Figure 3a,b, and the results are shown in Figure 3c,d. As can be seen from Figure 3c, the *I_dark_* value of PbS films grown on quartz substrates is proportional to the annealing temperature when the annealing temperature is less than or equal to 600 °C. This trend arises from annealing-induced recrystallization, which reduces grain boundaries and lowers potential barriers to carrier transport [22]. However, as the temperature surpasses 600 °C, the *I_dark_* value decreases due to sublimation of the in this temperature range (as illustrated in Figure 2j) and excessive oxidation. Figure 3d illustrates that the *R* and D* values of PbS films grown on quartz substrates increase with increasing annealing temperature when the temperature is less than or equal to 650 °C. At an annealing temperature of 650 °C, sample S650 achieved its maximum *R* and D* values, measuring 1.67 A W^−1^ and 1.22 × 10^10^ cm Hz^1/2^ W^−1^, respectively. Further increases in the annealing temperature caused a sharp decline in the *R* and D* values for sample S700, nearly reaching zero. The increases in the *R* and D* values were mainly attributed to the combination of improved crystal quality due to recrystallization at high temperatures, repair of internal defects in PbS, and oxygen sensitization of PbS [22]. A detailed discussion on the oxygen sensitization mechanism for PbS films will follow. The diminished *R* and D* values in sample S700 likely stemmed from excessive oxidation.

As mentioned in the “Introduction” section, the precise physical mechanism behind the optoelectronic sensitization of PbS films remains a topic of ongoing investigation. However, there is unanimous consensus regarding the impact of oxygen on enhancing the performance of thin PbS films. Previous studies have highlighted the substantial influence of oxygen content on the photosensitive properties of PbS films [11]. Researchers have demonstrated that oxygen can be used as a p-type doping impurity in PbS films [18,30]. Notably, Harada et al. [31] conducted annealing experiments on n-type PbS films in an O_2_ atmosphere, successfully converting them into p-type PbS films. In this study, we build upon the experimental results obtained from PbS thin films grown on quartz substrates to delve deeper into the sensitization mechanism of PbS thin films. It is essential to emphasize the robust bonding between the atoms of the quartz substrate and their excellent temperature stability. These qualities ensure that the quartz substrates remain structurally stable within the temperature range of our sensitization study, thus preventing any potential influence from the diffusion of oxygen elements originating from the substrates on the properties of the PbS thin film.

**Figure 4:**
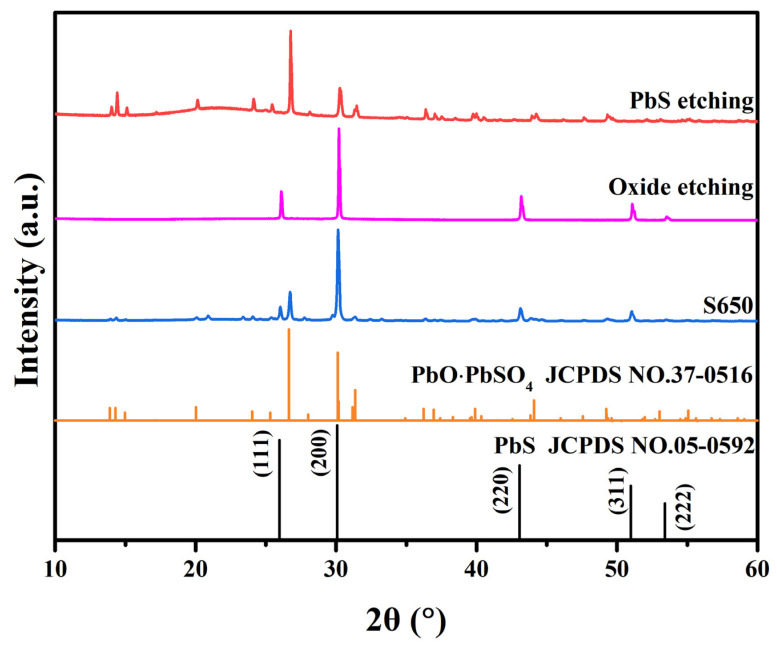
XRD patterns of sample S650 before and after chemical etching.

To further elucidate the mechanism of oxide effects on the optoelectronic properties of the annealed PbS films, sample S650 was partially chemically etched with photoresist protection. The PbS was etched with an aqueous solution of K_3_[Fe(CN)_6_] and Na_2_S_2_O_3_·5H_2_O at a mass ratio of 1:1 (PbS etching), while the oxide layer was etched with a solution of ammonium acetate (CH_3_COONH_4_) at a mass concentration of 20% (oxide etching). Figure 4 shows the XRD patterns of the PbS films before and after chemical etching. From the XRD patterns, only the diffraction peak of PbS was observed in the oxide-etched PbS film (JCPDS NO. 05-0592). Meanwhile, in the XRD patterns of the PbS film after PbS etching, only the diffraction peak of PbO·PbSO_4_ was observed (JCPDS NO.37-0516). This outcome underscores the effectiveness of the chosen chemical etching process in eliminating both oxide and PbS components. Notably, no PbSO_4_ diffraction peaks were identified in the PbS etching patterns for sample S650, likely due to the minimal PbSO_4_ content remaining in the films after chemical etching, falling below the detection threshold of XRD measurements.

**Figure 5:**
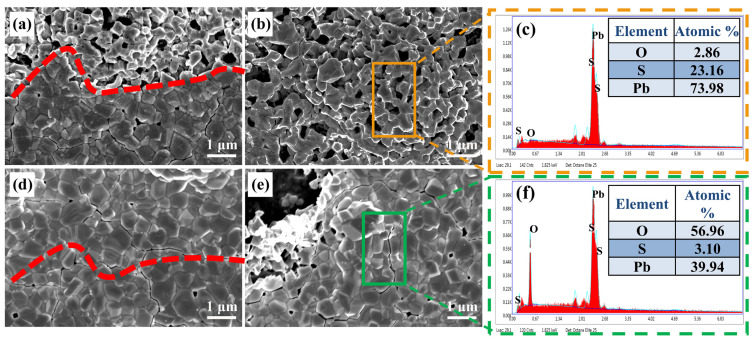
Diagrams of sample S650 after oxide etching: (**a**) interface; (**b**) etching section; (**c**) EDS. Diagrams of sample S650 after PbS etching: (**d**) interface; (**e**) etching section; (**f**) EDS. The red dotted line is the boundary between etching with and without photoresist protection. Above the red dotted line is the area without photoresist protection, below the red dotted line is the area with photoresist protection. Orange and green lines are EDS spot scanning areas.

Figure 5 shows the FESEM and EDS images of sample S650 after etching. As can be seen in the bottom areas of Figure 5a (below the red dashed line), the surface of the photoresist-protected film is distributed with large pebble-like grains. Meanwhile, in the areas without photoresist protection (upper areas of Figure 5a,b), small gravel-like grains are distributed, and the grains are interconnected to form a honeycomb-like network structure. In the EDS diagram of Figure 5c, the elemental O shows an extremely low content, indicating that the honeycomb structure is made up of PbS grains. Nevertheless, it can be seen from Figure 5d that the same pebble-like grains are present in both the etched PbS region (the upper region of Figure 5d) and the photoresist-protected region (the lower region of Figure 5d), and the higher elemental O content in Figure 5f indicates that the mixed solution of K_3_[Fe(CN)_6_] and Na_2_S_2_O_3_·5H_2_O did not etch the oxide phase. In contrast, only the diffraction peak of the PbO·PbSO_4_ phase can be observed in the XRD pattern of PbS etching in Figure 4, indicating that the pebble-like grains on the surface are the morphology of the oxide, and the oxide covers the PbS in a complementary honeycomb network structure. The larger void observed in Figure 5e was due to the collapse of the oxide phase as the bottom PbS was etched and lost its support. The FESEM results indicate that although the diffusion of O_2_ was deep during the high-temperature annealing at 650 °C, it was insufficient to completely block the PbS grains, and the internal PbS was still interconnected in a honeycomb network structure.

**Figure 6:**
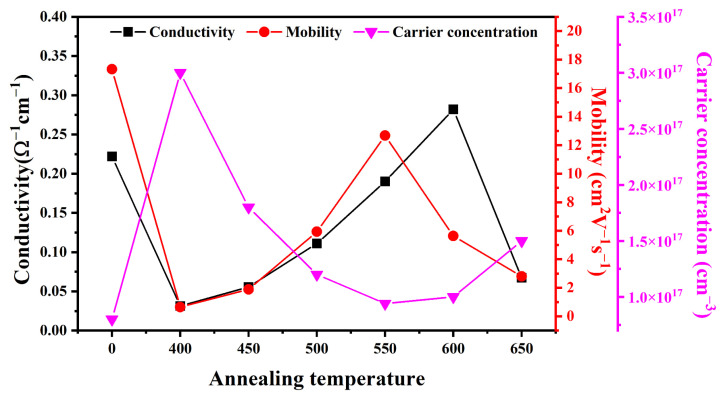
Hall measurements of carrier concentration, mobility, and conductivity versus annealing temperature.

To further elucidate the variation in electrical properties with annealing temperature, Hall measurements were employed to characterize the carrier concentration, mobility, and conductivity of the as-grown PbS films and those annealed at different temperatures. The results are shown in Figure 6. It is worth noting that the electrical properties of sample S700 were no longer detectable by Hall measurements, as it had become nearly isolated. As shown in Figure 6, the carrier concentration of sample S400 increased significantly from 8 × 10^16^ cm^−3^ to 3 × 10^17^ cm^−3^ compared to the as-grown film. However, this increase was accompanied by a significant decrease in both conductivity and mobility. As the annealing temperature gradually increased from 400 °C to 650 °C, the conductivity and mobility exhibited an initial rise, followed by a subsequent decline, while the carrier concentration displayed the opposite trend. Lower annealing temperatures cause some of the introduced oxygen ions not to be activated to the proper lattice positions, leading to a significant increase in shallow defects in the material, which releases more free carriers at room temperature [32]. Diffusion of oxygen improves the crystallinity of the material, passivating some internal defects and introducing new defects, affecting carrier trapping and scattering, thus altering the carrier mobility [33]. The increase in carrier concentration at higher annealing temperatures can be attributed to the diffusion of oxygen atoms into unoccupied S vacancies, passivating the dislocations and reducing carrier capture. The increase in grain size and the reduction in grain boundaries also contribute to the increase in mobility [13]. Notably, at an annealing temperature of 550 °C, the carrier concentration and mobility reached their minimum and maximum values of 9.4 × 10^16^ cm^−3^ and 12.68 cm^2^ V^−1^ s^−1^, respectively. Nevertheless, the conductivity reached its maximum value of 0.282 Ω^−1^ cm^−1^ when the annealing temperature was 600 °C.

**Figure 7:**
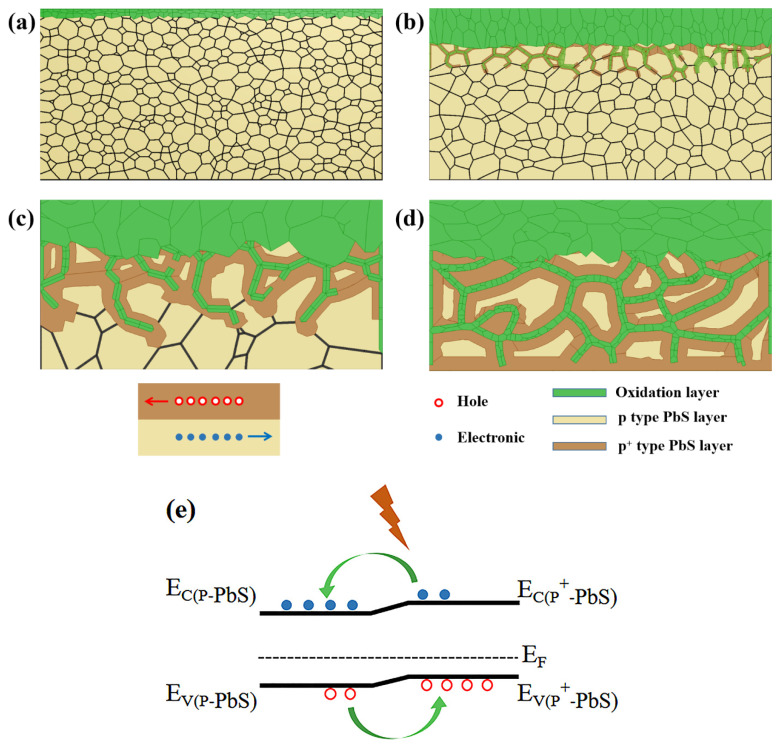
Schematic diagram of the 3D network model of the sensitization mechanism of PbS thin films: (**a**) Early sensitization. (**b**) Under-sensitization. (**c**) Proper sensitization. (**d**) Over-sensitization. (**e**) Band diagram of the p-PbS/p^+^-PbS structure under IR excitation.

Based on the microstructural evolution, composite phase, and photoelectric change of the annealed and etched PbS films, a new three-dimensional network model of the sensitization mechanism of PbS thin films based on the charge separation model is proposed, as illustrated in Figure 7. To elucidate the evolving internal details of the film at distinct sensitization stages, the as-grown PbS film is described as consisting of a number of pale yellow pebble-like microcrystals with varying sizes. The as-grown films are p-type PbS, with a low carrier concentration, high electrical conductivity, and poor photosensitivity due to the large number of defects in the films. Based on the 3D carrier separation model, our experimental results can be coherently interpreted as follows: At the initial stage of sensitization (sample S400), as shown in Figure 7a, the annealing temperature is low, and O_2_ not only reacts with PbS grains on the surface to form oxides (Figure 2b), which reduces the conductivity, but also dopes the film with p-type PbS, resulting in a significant increase in the carrier concentration. As a result, the carrier concentration of sample S400 (3 × 10^17^ cm^−3^) is 375% of the carrier concentration of the as-grown sample (8 × 10^16^ cm^−3^). Nevertheless, the mobility decreases due to the enhanced scattering effect of impurities [34]. In this case, both the *R* and D* values are small, leading to poor photosensitivity.

As the sensitization proceeds with increasing annealing temperature (samples S450~S550), significant recrystallization of the PbS grains occurs (Figure 2c–e). This process reduces grain boundaries and decreases potential barrier heights for carrier transport, resulting in increased conductivity. Simultaneously, defects within the film are repaired during the annealing process, resulting in an increase in carrier mobility. It is noteworthy that at higher annealing temperatures, the enhanced permeability of the elemental O leads to more O doping, and p^+^-type layers are formed in the upper layer of the film between the PbS grains. However, as the annealing temperature is still low at this stage, the p^+^-type layers between the grains are small and dispersed, and the level of p^+^-type layers in the films is insufficient to establish a continuous 3D interweaving network structure, as shown in Figure 7b. The limited impact of oxygen doping, particularly at lower annealing temperatures, leads to a decrease in carrier concentration for PbS films, where point defects (i.e., impurities and vacancies) are the primary carriers. This effect is further pronounced with the absence of oxygen doping in the lower regions, resulting in improved photosensitivity. This enhancement can be primarily attributed to defect repair through recrystallization and the reduction in grain boundaries.

Upon reaching the optimal sensitization temperature (sample S650), further enhanced recrystallization of the PbS grains occurs, accompanied by extensive defect repair. At this stage, oxygen diffuses along the boundaries of PbS grains into the interior of the PbS films. The p^+^-type PbS layers begin to connect and form a 3D interweaving network structure. Simultaneously, a significant amount of oxide is generated, but the formed oxide layer does not completely block the connection between PbS grains due to the coarse grain interfaces. In this scenario (Figure 7c), photogenerated electron–hole pairs were generated in both p-type PbS and p^+^-type PbS under IR laser excitation at a wavelength of 1550 nm. After an effective contact between p^+^-type PbS and p-type PbS, the p^+^-type PbS 3D network structure distributed in the interface of the PbS crystalline grains provides the conducting channels for the photogenerated holes, while the interconnected p-type PbS grains serve as the electron-conducting channels. This charge transfer enables the existence of a “built-in electric field”, akin to that of a p-n junction, facilitating charge transport. The presence of the “built-in electric field” allows charges to be directed through the Fermi energy level, accelerating carrier separation. The carrier transport process is schematically illustrated in Figure 7e. Under bias, photogenerated electrons and holes are transported through p-type PbS grains and p^+^-type PbS 3D network conducting channels, respectively. This results in effective electron–hole separation and reduced electron–hole recombination, which then trigger the IR response. In this case, an explanation based on the p^+^-p charge separation junction mechanism of PbS IR detection can be proposed. This effective separation of electrons and holes via the p^+^-p junction prolongs the lifetime of photogenerated carriers and yields high photocurrent, low dark current, and strong photosensitivity, ultimately reaching the maximum detectivity of the PbS detector. However, when the annealing temperature is too high (over-sensitization), the diffusion depth of O is strongly enhanced and it spreads along the grain boundaries throughout the PbS film. This scenario, as illustrated in Figure 7d, leads to the replacement of the oxide layer and interfaces between PbS grains with oxide, entirely obstructing the interconnection between the PbS grains. Consequently, the resistance becomes notably high (exceeding 18 MΩ), and the dark current substantially decreases, resulting in the disappearance of the IR response.

## 4. Conclusions

In summary, we synthesized large-area PbS films consisting of crystalline particles of different sizes directly on quartz substrates using the CBD method. We undertook a systematic investigation to elucidate the mechanism of oxygen sensitization in PbS detectors by varying the annealing temperature. As the annealing temperature increased, the SEM surface morphology results revealed a gradual increase in surface grain size, attributed to oxidation. Concurrently, the XRD patterns exhibited an increased number of oxide-induced diffraction peaks, indicating a heightened degree of oxidation. Notably, at an annealing temperature of 650 °C, the D* value of the PbS detector reached its maximum value of 1.22 × 10^10^ cm Hz^1/2^ W^−1^. Drawing from the observed microstructural evolution, composite phase, and photoelectric changes under various sensitization processes, a p^+^-p 3D network transport model was proposed to elucidate the charge separation mechanism of PbS IR detection. The 3D network conducting model provides a robust explanation for the charge separation mechanism governing PbS IR photoconductive detection.

## Data Availability

Not applicable.
